# The Usefulness of Rapid Diagnostic Tests in the New Context of Low Malaria Transmission in Zanzibar

**DOI:** 10.1371/journal.pone.0072912

**Published:** 2013-09-04

**Authors:** Delér Shakely, Kristina Elfving, Berit Aydin-Schmidt, Mwinyi I. Msellem, Ulrika Morris, Rahila Omar, Xu Weiping, Max Petzold, Bryan Greenhouse, Kimberly A. Baltzell, Abdullah S. Ali, Anders Björkman, Andreas Mårtensson

**Affiliations:** 1 Malaria Research, Department of Medicine Solna, Karolinska Institutet, Stockholm, Sweden; 2 Division of Global Health (IHCAR), Department of Public Health Sciences, Karolinska Institutet, Stockholm, Sweden; 3 Zanzibar Malaria Control Programme, Ministry of Health, Zanzibar, Tanzania; 4 Department of Infectious Diseases, Institute of Biomedicine, University of Gothenburg, Gothenburg, Sweden; 5 Centre for Applied Biostatistics, Sahlgrenska Academy, University of Gothenburg, Gothenburg, Sweden; 6 Department of Medicine, University of California San Francisco, San Francisco, California, United States of America; 7 Department of Family Health Care Nursing, University of California San Francisco, San Francisco, California, United States of America; 8 Department of Medicine, Kungälv Hospital, Kungälv, Sweden; Menzies School of Health Research, Australia

## Abstract

**Background:**

We assessed if histidine-rich-protein-2 (HRP2) based rapid diagnostic test (RDT) remains an efficient tool for Plasmodium falciparum case detection among fever patients in Zanzibar and if primary health care workers continue to adhere to RDT results in the new epidemiological context of low malaria transmission. Further, we evaluated the performance of RDT within the newly adopted integrated management of childhood illness (IMCI) algorithm in Zanzibar.

**Methods and Findings:**

We enrolled 3890 patients aged ≥2 months with uncomplicated febrile illness in this health facility based observational study conducted in 12 primary health care facilities in Zanzibar, between May-July 2010. One patient had an inconclusive RDT result. Overall 121/3889 (3.1%) patients were RDT positive. The highest RDT positivity rate, 32/528 (6.1%), was found in children aged 5–14 years. RDT sensitivity and specificity against PCR was 76.5% (95% CI 69.0–83.9%) and 99.9% (95% CI 99.7–100%), and against blood smear microscopy 78.6% (95% CI 70.8–85.1%) and 99.7% (95% CI 99.6–99.9%), respectively. All RDT positive, but only 3/3768 RDT negative patients received anti-malarial treatment. Adherence to RDT results was thus 3887/3889 (99.9%). RDT performed well in the IMCI algorithm with equally high adherence among children <5 years as compared with other age groups.

**Conclusions:**

The sensitivity of HRP-2 based RDT in the hands of health care workers compared with both PCR and microscopy for P. falciparum case detection was relatively low, whereas adherence to test results with anti-malarial treatment was excellent. Moreover, the results provide evidence that RDT can be reliably integrated in IMCI as a tool for improved childhood fever management. However, the relatively low RDT sensitivity highlights the need for improved quality control of RDT use in primary health care facilities, but also for more sensitive point-of-care malaria diagnostic tools in the new epidemiological context of low malaria transmission in Zanzibar.

**Trial registration:**

ClinicalTrials.gov NCT01002066

## Introduction

Zanzibar has recently undergone a rapid transition from high to low transmission of *Plasmodium falciparum* malaria following wide scale, high coverage implementation of combined malaria control interventions [1]. In this new epidemiological context, efficient malaria case detection and targeted malaria treatment with artemisinin-based combination therapy (ACT) to fever patients with parasitologically confirmed malaria infection is critical. Therefore, Zanzibar introduced *P. falciparum* specific rapid diagnostic testing (RDT), based on antigen detection of histidine-rich protein 2 (HRP2), for confirmatory malaria diagnosis of fever patients in all public health care facilities previously not equipped with microscopy service [2]. Importantly, malaria RDT has also been recently incorporated in the local version of the integrated management of childhood illness (IMCI) guidelines of 2009 [3], [4]. These guidelines are used for management of illness in children aged 2–59 months in low and middle-income countries. The performance of RDT as an integrated tool in IMCI for improved childhood fever management in Africa and Zanzibar in particular has previously not been evaluated.

There are concerns regarding the usefulness of RDT, both with regards to case detection with differences in test performances in various epidemiological settings and to adherence to negative test results among health care workers [5], [6], [7], [8], [9]. However, we have previously shown that HRP2 based RDT aided diagnosis of fever patients in primary health care facilities in Zanzibar was efficient for *P. falciparum* detection when compared with blood smear (BS) microscopy with a sensitivity of 92% and a specificity of 88%; moreover, RDTs resulted in improved adequate treatment and health outcomes [10]. Importantly, the previous study was conducted when *P. falciparum* malaria was responsible for approximately 30% of febrile illness in primary health care facilities in Zanzibar [10], [11]. This is in marked contrast to the present situation, when overall <3% of fever patients have confirmed *P. falciparum* infection [12].

Based on the above, three critical scientific questions can be identified: Firstly, does HRP2 based RDT remain an efficient tool for *P. falciparum* detection in the new epidemiological context in Zanzibar? This should be evaluated not only against conventional BS microscopy but also against highly sensitive PCR as gold standard, considering that the relative importance of parasitemias below the detection limit of RDT/microscopy may increase in low transmission settings [13], [14], [15]. Secondly, is malaria RDT, as an integrated part of the recently adopted local version of the IMCI algorithm, a reliable tool to both identify and rule out malaria infection in children below five years of age? Thirdly, and importantly, do primary health care workers continue to adhere to RDT results when over 95% of the tests are negative and alternative diagnostic tools for fever management are scarce?

The aim of this study was therefore to evaluate the usefulness of HRP2 based RDT in the hands of primary health care workers, including its performance within the local version of the IMCI algorithm, for *P. falciparum* case detection, and adherence to test results among fever patients in the new epidemiological context of low malaria transmission in Zanzibar.

## Methods

### Study Design, Area and Sites

This work was a health facility based observational study conducted in twelve primary health care facilities, six each in North A (Unguja Island) and Micheweni (Pemba Island) districts of Zanzibar, between May and July 2010**.** None of these health facilities were part of our previous RDT study from Zanzibar [10]. The two study districts are mainly rural with approximately 100,000 inhabitants each. *P. falciparum* is the predominant malaria species and *Anopheles gambiae* complex the main vector. Malaria transmission has historically been stable with peaks related to the seasonal rainfalls in March to May and October to December. Following a successful malaria control programme the BS/RDT positivity rate among fever patients has markedly declined during the last decade in Zanzibar to reach 0.6% and 2.2%, respectively, in North A and Micheweni districts in 2009 [12].

Public health care in the study area is delivered through twelve primary health care units (PHCU) and one primary health care centre (PHCC) per district. The PHCUs provide basic out-patient care including malaria RDT for fever management, whereas the PHCCs in addition to basic out-patient care also have facilities for in-patient care and laboratory services, e.g. routine use of microscopy for confirmatory malaria diagnosis.

The twelve study sites were purposely selected, i.e. one PHCC and five PHCU from each district. This was done to ensure an adequate manpower capacity, with at least two health workers available per study site during the conduct of the trial, and to provide a balanced geographical distribution of the study sites in the respective districts.

### Study Staff and Pre-study Training

A total of 33 nurses and clinical officers with formal prescription rights from Zanzibar Ministry of Health were responsible for the outpatient care in the twelve study sites. All study staff members were previously trained in malaria case management by the Zanzibar Malaria Control Programme (ZMCP). Based on previous IMCI training, the health workers received either a six-day refresher course or an eleven-day full course on the recently adopted IMCI guidelines in Zanzibar in which malaria RDT has been integrated in the algorithm [3]. In addition to the IMCI training and prior to the study start, all study staff members also received a one-week study specific training including good clinical practice, ethical considerations and provision of informed consent, data recording in case record forms (CRF), blood sampling for BS and filter papers as well as performance and interpretation of RDT (Paracheck Pf®) according to the manufacturer’s instructions. During the pre-study training the study staff members were encouraged to adhere to the IMCI guidelines for management of children below five years of age and the national malaria guidelines, which recommend anti-malarial medicines to be prescribed based on laboratory confirmation of malaria [2]. However, they were free to manage patients according to their own clinical judgement during the conduct of the trial. Prior to the first enrolment a pilot study with “dummy runs” was conducted in all participating study sites.

All health workers received a fixed monthly salary as top ups for their study participation according to standard practice in Zanzibar, whereas study participants did not receive any incentives.

### Patient Enrolment and Management

Patients were eligible if aged ≥2 months and presenting at the study sites with fever, i.e. measured axillary temperature of ≥37.5°C or a history of fever during the preceding 24 hours, and willing to provide written informed consent (proxy consent from parents/guardians for children) to participate in the study. Patients were excluded and referred in case of any symptoms of severe disease or danger signs. Patients were recruited Monday to Friday between eight am to four pm by the study health worker on duty in the respective study sites. Children aged 2–59 months, hereafter referred to as “<5 years”, were managed according to the local IMCI guidelines. The study included a single visit for each participant.

Upon enrolment a sealed envelope was opened containing a study reference number and information on whether the individual had been randomly selected for additional blood sampling (see below). This was followed by a capillary finger prick for performance of RDT. All RDT positive patients and a pre-defined computer generated random sample (www.randomization.com) of 20% of all study participants were subjected to additional capillary blood sampling for malaria microscopy (thick BS) and PCR (approximately100 µL spotted on a Whatmann 3 MM filter paper).

Basic demographical data, axillary temperature (measured with a digital thermometer), history of fever in the preceding 24 hours, other symptoms, previous anti-malarial treatment (in case of pregnancy use of intermittent presumptive treatment (IPTp)), whether the patient slept under an insecticide treated nets/long lasting insecticidal nets (ITN/LLIN) the night before study enrolment and travel history (defined as having spent at least one night outside the home Shehia, i.e. the smallest administrative unit in Zanzibar, in the last 28 days) were recorded in a CRF together with information on RDT-result and clinical management.

The RDT results were provided directly to the patients by the study staff. Anti-malarial medicines according to national guidelines, i.e. artesunate-amodiaquine (first-line) or oral quinine (second-line), antibiotics (cotrimoxazole, ampicillin, amoxicillin or erythromycin) and antipyretics (e.g. paracetamol) were available free of charge to all study participants. Patients and guardians were encouraged to return if the clinical condition deteriorated or if the fever persisted.

In case malaria parasitemia was first detected by first BS microscopy reading and missed by RDT diagnostic (see below), the result was reported to the health facility and the patient was traced and offered treatment if he/she did not receive anti-malarial medicines at the time of the study visit.

All CRFs were collected by trained health staff at the end of each day and stored centrally in Zanzibar. During the conduct of the study, no results were disclosed to the health workers. However, after study completion, preliminary results were disseminated to all health workers in both districts.

### Laboratory Methods

#### RDT

We used Paracheck Pf (Orchid Biomedical Systems, Goa, India) detecting *P. falciparum* specific HRP2 antigen, which by the time of trial was the RDT device deployed by ZMCP. The tests were performed and interpreted according to the manufacturer’s instructions. Very faint bands at the test line position were to be assessed as a positive test result. Band intensity was not recorded.

#### Blood smear microscopy

BS were prepared on site, stored in slide boxes and transported daily to the district central level, where all slides were stained with 5% Giemsa for 30 minutes and examined under oil immersion (×100 magnification) by two independent and experienced microscopists blinded to both the RDT and each other’s microscopy result [16]. Asexual parasite densities were calculated against 200 white blood cells (WBC), assuming 8,000 WBC per microliter of blood. BS were defined negative if no parasites were found after examining 100 high power microscopy fields. BS with discordant results between the two readers, i.e. positive versus negative, difference in species diagnosis or a difference of >50% in parasite density, were re-examined by a third expert microscopist at Karolinska Institutet, Stockholm, Sweden. The mean of the two most concordant parasite counts were used for calculating the final parasite density [17]. Moreover, all BS from patients with discrepancies between RDT and PCR results, RDT and BS results or PCR and BS results were subjected to a third decisive microscopy reading at Karolinska Institutet.

#### Filter paper

The blood samples (approximately 100 µL) collected on filter papers (Whatmann 3 MM) were dried thoroughly, put in individual zipped plastic bags containing desiccant and stored in room temperature (<25°C) in Zanzibar until completion of the study and then transported to Karolinska Institutet for molecular analyses.

#### DNA extraction

DNA was extracted from three filter paper punches (Ø 3 mm) using a modified version of the ABI 6100 Nucleic Acid Prep Station protocol (Applied Biosystems, Fresno, CA) [18]. In samples with discrepancies between RDT and PCR results, DNA was re-extracted from the filter papers using Chelex-100 [19].

#### DNA extraction quality control

The presence of human DNA was analysed by real-time PCR in case of PCR-negative results in RDT positive samples [20].

#### Parasite detection by PCR

For the purpose of an ongoing genotyping project all RDT positive samples were analysed with three previously described *P. falciparum* specific nested PCR methods targeting the four antimalarial drug resistance markers *P. falciparum* multidrug resistance gene 1 (*pfmdr1*) N86Y, Y184F, D1246Y and *P. falciparum* chloroquine resistance transporter gene (*pfcrt*) K76T [21], [22]. The RDT negative samples were screened in duplicate for human plasmodial infection with an 18s real-time PCR [23] Samples with a cycle threshold (Ct) value <42 were selected for species identification. Samples with discrepant RDT and PCR results were re-extracted and subjected to a confirmatory nested PCR analysis targetin*g Plasmodium* cytochrome b [19]. PCR positivity was defined as a positive PCR result that could be validated by at least one other PCR method or by parasite detection by microscopy.

#### Analysis of P. falciparum HRP2 deletion

All samples with a negative RDT but a positive microscopy and/or PCR result were subjected to PCR analysis of HRP2 deletion according to a previously described protocol [24].

### Adherence to RDT Results

Assessment of adherence to RDT results was done based on a comparison between the reported RDT outcome and prescription of anti-malarial drugs. Adherence was defined as prescription and absence of prescription of anti-malarial drug (first or second line) in RDT positive and negative patients, respectively.

### Sample Size Calculation, Study Endpoints, Data Management and Statistical Analysis

The sample size calculation was based on the primary endpoint, i.e. adherence to malaria RDT results, with the assumption that approximately 10% of the RDT negative patients are prescribed ACT in Zanzibar [10]. The minimal sample size for independent observations would be 864 patients when requiring a maximum length of +/−2% for a 95% confidence interval (CI). Considering a potential clustering effect on health facility level we calculated that a sample of minimum 3054 patients would allow for an intra-cluster coefficient of slightly over 0.01 and still fulfil the given level of precision.

Secondary endpoints included sensitivity, specificity, positive and negative predictive values of HRP2 based RDT for *P. falciparum* detection compared with both PCR and BS microscopy; performance of malaria RDT as an integrated part of the local IMCI algorithm in Zanzibar (assessed as a sub-group analysis of sensitivity, specificity, positive and negative predictive values in children aged <5 years compared with other age groups); and antibiotic and antipyretic treatment across age-groups, i.e. <5, 5–14 and >14 years, among RDT positive and negative patients.

Data were double entered in CSPro, validated using Microsoft Excel and subsequently exported to STATA 12 software where all statistical analyses were performed. All frequencies, proportions and odds ratios (ORs) were calculated with 95% CIs and corresponding p-values, as appropriate. Adjustments for clustering on health facility level were done using mixed effect models.

To account for the fact that blood sampling for BS microscopy and PCR among RDT negative patients only included a random sample of 20%, we multiplied the absolute number of observations in these groups with a factors of 5.14 and 5.01 in all calculations of RDT sensitivity and specificity against PCR and microscopy, respectively. The corresponding confidence intervals were, however, based on the true sample size. Statistical significances were stated at the 5% level.

### Ethical Considerations

The study was conducted in accordance with the principles stated in the latest version of the Declaration of Helsinki and Good Clinical Practice [25]. It was approved by the Zanzibar Medical Research Ethics Committee (ZAMREC) and the Regional Ethics Committee, Stockholm, Sweden. The study is registered on ClinicalTrials.gov with study identifier “NCT01002066”.

## Results

**Figure 1:**
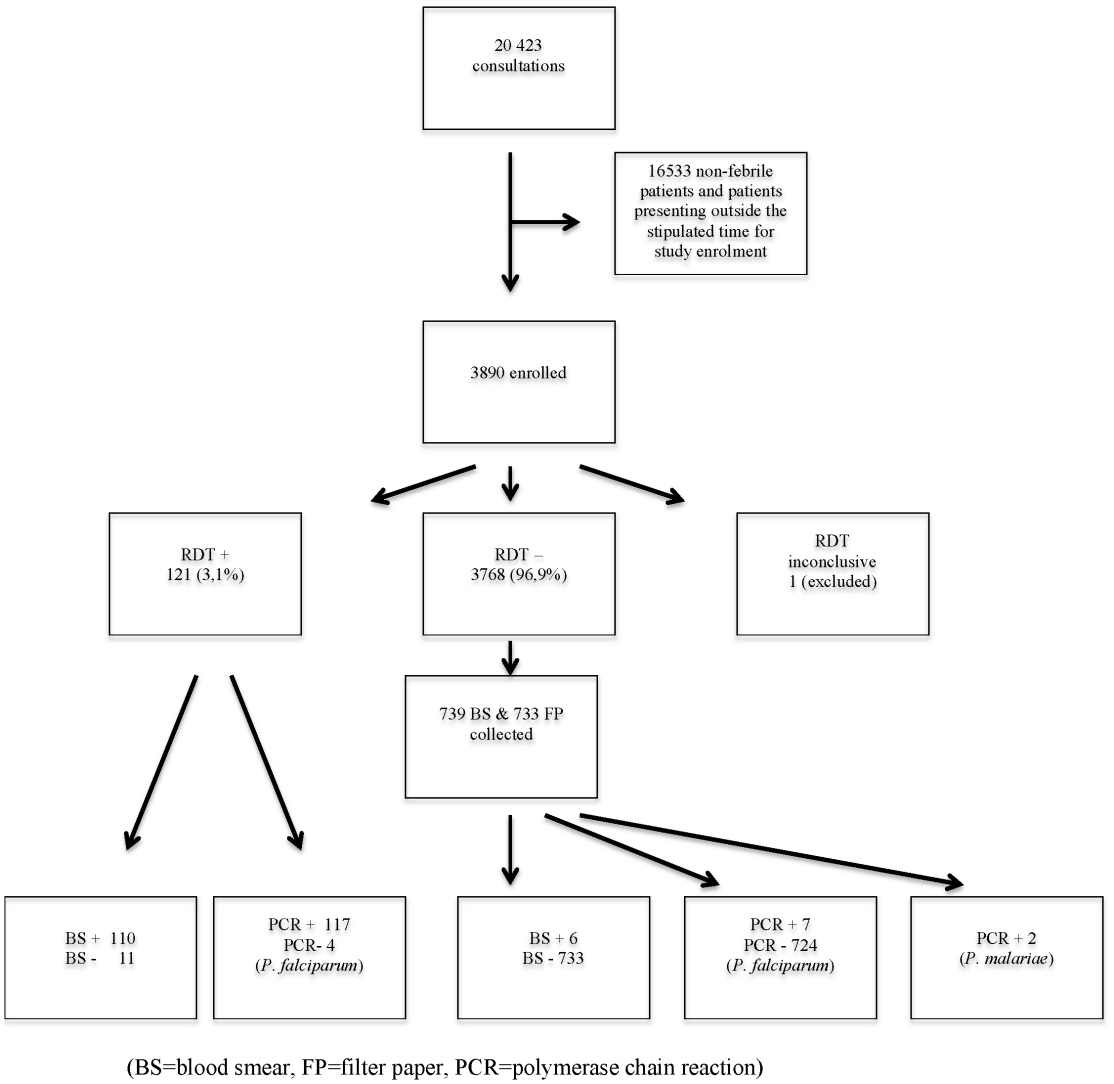
Study flow chart. (BS = blood smear, FP = filter paper, PCR = polymerase chain reaction.).

The flow of patients through the trial is outlined in Figure 1 and baseline characteristics of the RDT positive and negative groups are presented in Table 1. Among the 3890 patients enrolled one was excluded due to an inconclusive RDT result. Overall 121/3889 (3.1%) patients were RDT positive. The highest RDT positivity rate, i.e. 32/528 (6.1%), was found in children aged 5–14 years. There was an uneven distribution of RDT positivity rates between the two districts, i.e. North A 42/2225 (1.9%) and Micheweni 79/1665 (4.7%), and also between their respective study sites with 53 (67.1%) of all RDT positive patients in Micheweni district reported from Tumbe PHCU only. RDT positivity was statistically significantly associated with absence of ITN/LLIN use [OR 4.78 (95% CI 3.24–7.02)] and history of travel within the last 28 days [OR 3.27 (95% CI 2.05–5.07)] (Table 1).

**Table 1:** Baseline characteristics.

		RDT +	RDT −	Total (N)
**District**	North A	42 (34.7%)	2182 (57.9%)	2224
	Micheweni	79 (65.3%)	1586 (42.1%)	1665
**Age**	5 y	36 (29.8%)	1786 (47.4%)	1822
	5–14 y	32 (26.4%)	496 (13.2%)	528
	>14 y	53 (43.8%)	1472 (39.1%)	1525
	Data missing	0	14 (0.3%)	14
**Sex**	Male	65 (53.7%)	1611 (42.8%)	1676
	Female	56 (46.3%)	2156 (57.2%)	2212
	Data missing	0	2 (100%)	2
**Pregnancy**		3 (2.5%)	87 (2.3%)	90
		**RDT +**	**RDT −**	**P-value**
**Mean axillary temp.°C**		38.3	37.4	<0.001
**Proportion withaxillary temp. ≥37.5**°**C**		74 (66.7%)	1465 (41.5%)	<0.001
**Data missing**		10 (8.3%)	240 (6.4%)	0.350
**ITN/LLIN use**		65 (53.7%)	3193 (84.7%)	<0.001
**Travel history**	≤28 days	30 (24.8%)	356 (9.5%)	<0.001
	Domestic	21 (17.4%)	325 (8.6%)	0.003
	Abroad	9 (7.4%)	31 (0.8%)	<0.001

Domestic = History of travel within Zanzibar.

Abroad = History of travel outside Zanzibar.

ITN = Insecticide-treated nets.

LLIN = long-lasting insecticidal nets.

The distribution of positive RDT, PCR and BS results as well as microscopy determined parasite densities across age groups are shown in Table 2.

**Table 2:** Distribution of *P. falciparum* positive rapid diagnostic test (RDT), polymerase chain reaction (PCR) and blood smear (BS) microscopy results as well as microscopy determined parasite density across age groups.

	Age groups			Total
	<5 y n = 1822	5–14 y n = 528*	>14 y n = 1525*	
RDT +	36 (2.0%)	32 (6.1%)	53 (3.5%)	121
PCR +	34 (1.9%)	34 (6.4%)	56 (3.7%)	124
BS +	31 (1.7%)	33 (6.3%)	52 (3.4%)	116
Geometric mean parasite density/µL (range)	13815 (50–597,500)	14172 (12–782,400)	3739 (6–184,500)	

* Data on specific age categories (5–14 y and >14 y) were missing from14 patients.

The RDT sensitivity, specificity, positive and negative predictive values against PCR and BS microscopy are presented in Table 3. There was no significant difference in RDT performance in children aged <5 years, i.e. tested and managed within the IMCI algorithm, and other age groups (data not shown).

**Table 3:** Rapid diagnostic test (RDT) sensitivity, specificity, positive and negative predictive value against polymerase chain reaction (PCR) and blood smear (BS) microscopy.

	PCR +	PCR –	Total	BS+	BS−	Total
RDT +	117	4	121	110	11	121
RDT –	7*	726*	733	6**	733**	739
Total	124	730	854	116	744	860
Sensitivity	76.5% (95% CI 69.0– 83.9%)			78.6% (95% CI 70.8–85.1)		
Specificity	99.9% (95% CI 99.7– 100%)			99.7% (95% CI 99.5–99.9%)		
Positive predictive value	96.7% (95% CI 91.8– 99.1%)			91.7% (95% CI 84.3–95.4%)		
Negative predictive value	99.0% (95% CI 98.0– 99.6%)			99.2% (95% CI 98.8–99.5%)		

* For calculations of sensitivity and specificity the absolute numbers in the RDT negative group were multiplied with a factor of 5.14 to account for that only a sub-sample, i.e. 733 of 3768, in this group were subjected to blood sampling on filter paper for PCR.

** For calculations of sensitivity and specificity the absolute numbers in the RDT negative group were multiplied with a factor of 5.01 to account for that only a sub-sample, i.e. 739 of 3768, in this group were subjected to blood sampling for BS microscopy.

There were four and eleven RDT positive patients with negative PCR and BS results, respectively. Importantly seven patients had both positive RDT and PCR verified *P. falciparum* infection, but BS microscopy could not detect parasitemia. Some, 6/739 (0.8%) RDT negative patients had detectable parasitemia by BS microscopy; all but one aged ≥5 years, of whom three were from Tumbe PHCU (Micheweni). Four of these six patients had high parasite densities, i.e. 3573, 14420, 25779, 50190 parasites/µL, respectively, whereas the remaining two had low parasite counts of ten and 50 parasites/µL each. All six were also positive by PCR, five for *P. falciparum* and one for *P. malariae*. The *P. malariae* PCR positive sample had a low parasite density of ten parasites/µL, as determined by BS microscopy, i.e. insufficient to allow accurate species identification. Further PCR analysis could not detect *P. falciparum* HRP2 deletions in any of these samples. Four of the RDT negative patients had a positive first BS microscopy reading. They were all successfully traced at home and received treatment with artesunate-amodiaquine. None had developed symptoms/signs of severe malaria between day of study visit and time of active follow-up.

Prescription of anti-malarial medicines, antibiotics and antipyretics by RDT result and age group are shown in Table 4. All 121 RDT positive, but only 3/3768 (0.1%) of RDT negative patients, all aged >14 years, were prescribed anti-malarial medicines. Overall adherence to RDT results was thus 3886/3889 (99.9%). Among the 124 patients that were prescribed anti-malarial medicines, 119 (96.0%) received artesunate-amodiaquine, four (3.2%) quinine and the remaining one (0.8%) a combination of artesunate-amodiaquine and quinine.

**Table 4:** Prescription of anti-malarial medicines, antibiotics and antipyretics by rapid diagnostic test (RDT) result and age group.

Prescription	Age	RDT +	RDT −	P-value
**Anti-malarial medicines**	<5 y	36/36 (100%)	0/1786 (0%)	<0.001
	5–14 y	32/32 (100%)	0/496* (0%)	<0.001
	>14 y	53/53 (100%)	3/1472* (0.2%)	<0.001
**Antibiotics**	<5 y	20/36 (55.6%)	1032/1786 (57.8%)	>0.999
	5–14 y	8/32 (25.0%)	341/496* (68.8%)	<0.001
	>14 y	6/53 (11.3%)	804/1472* (54.6%)	<0.001
**Antipyretics**	<5 y	14/36 (38.9%)	437/1786 (24.5%)	0.053
	5–14 y	19/32 (59.4%)	278/496* (56.0%)	0.855
	>14 y	34/53 (64.2%)	894/1472* (60.7%)	0.669

* Data on specific age categories (5–14 y and >14 y) were missing from14 patients.

Antibiotics were prescribed to a total of 2218/3889 (57.0%) patients, of whom 24 (1.1%) received prescription of more than one antibiotic. Some 1338 (60.3%) received cotrimoxazole, 573 (25.8%) ampicillin/amoxicillin, 288 (13.0%) penicillin, 33 (1.5%) erythromycin, 29 (1.3%) metronidazole and 18 (0.8%) ciprofloxacin, respectively. RDT negative patients were overall statistically significantly more likely to receive antibiotics than RDT positive [OR 3.25 (95% CI 2.15–5.01)]. However, among children <5 years of age the rate of antibiotic prescription was similar between the groups, i.e. 57% and 58% in the RDT negative and RDT positive group, respectively. Among RDT positive patients, no statistically significant difference between children<5 years and patients aged ≥5 years was observed [OR 1.36 (95% CI 0.77–2.30)].”

Antipyretics were prescribed to 1689/3889 (43.4%) patients of whom 1558 (92.2%) received paracetamol and the remaining 122 (7.2%) non-steroidal anti-inflammatory drugs including acetylsalicylic acid. There was a balanced distribution of antipyretic prescription between the RDT positive and RDT negative group. However, children <5 years were overall statistically significantly less likely to receive antipyretics than patients aged ≥5 years [OR 0.22 (95% CI 0.19–0.26)].

## Discussion

The rapid transition from high to low malaria transmission recently observed in various areas of sub-Saharan Africa, including Zanzibar, constitutes a major challenge to ensure sustained high parasite-based *P. falciparum* diagnostic accuracy with malaria RDT among fever patients and high adherence to RDT results among health care workers. This is critical for safe and rational fever case management, including targeted ACT treatment to patients with confirmed malaria infection. The present study was therefore undertaken to assess if HRP2 based RDT remains an efficient tool for *P. falciparum* case detection among fever patients in Zanzibar, not only against BS microscopy but also against highly sensitive PCR, and if primary health care workers continue to adhere to RDT results in the new epidemiological context of low malaria transmission. Further, we scientifically evaluated, for the first time in Africa, the performance of RDT as an integrated part of the newly adopted IMCI algorithm in Zanzibar.

The observed diagnostic accuracy of HRP2 based RDT (Paracheck-Pf) for *P. falciparum* detection in this study indicates a relatively low sensitivity but a high specificity when compared with both BS microscopy and PCR. Since our previous report from Zanzibar [10], conducted when *P. falciparum* malaria was responsible for approximately 30% of fever episodes, the sensitivity of RDT against BS microscopy declined from 92% to 79%, whereas the corresponding specificity increased from 88% to 99%. The latter may reflect an overall reduced risk of repetitive malaria infections and remaining antigenemia from a previous malaria episode in the new low transmission context in Zanzibar. However, the relatively low sensitivity of Paracheck-Pf, i.e. below the reported sensitivity for *P. falciparum* detection for this RDT device in the most recent WHO/FIND evaluation [26], is a concern especially since the fact that similar observations have been documented in other low transmission areas [14]. Even lower RDT sensitivities than we observed have been reported from other studies i.e. 65% against BS microscopy in Rufiji district of Tanzania [27] and 69% against PCR in two Tanzanian hospitals [8].

The relatively low sensitivity may be at least partly due to sub-optimal RDT performance of individual health care workers, considering that approximately half of all RDT negative but BS positive cases were reported from the same health facility. This calls for improved systems for RDT supervision and quality control in primary health care facilities, but also for more sensitive point-of-care malaria diagnostic tools. Other factors, unrelated to health care workers performance, such as presence of *P. falciparum* HRP2 deletions and sequence variation as well as the so called “prozone effect”, need to be considered as a potential cause of the relatively low RDT sensitivity, particularly since the HRP2 based RDT failed to detect four cases of relatively high *P. falciparum* density, i.e. 3573, 14420, 25779 and 50190 parasites/µL, respectively [24], [28], [29]. Importantly, we did not detect any case of *P. falciparum* HRP2 deletions in these samples, which is in contrast to observation from Mali in West Africa [30]. However, HRP2 sequence variation was not analysed, since previous studies have not been able to link RDT sensitivity with sequence variation in clinical malaria infections with parasite densities ≥200 parasites/µL. Whether a prozone effect, defined as a false-negative result in an immunological reaction due to an excess of either antigens or antibodies, occurred in any of our samples could not be assessed retrospectively since blood was not available for serial dilution. Of note is though that the prozone phenomenon has previously been described primarily in patients with *P. falciparum* hyperparasitemia (≥250,000 parasites/µL) [31].

This study confirms our previous observations from Zanzibar of an excellent adherence to RDT results among primary health workers [10]. It is indeed reassuring that health care workers in Zanzibar continue to rely on RDT results in a context where overall 97 out of 100 tests are negative and scarce tests facilities for diagnosing alternative causes of fever causes are available. With our results in mind and considering the contradictory reports of adherence to RDT results from various levels of health care and epidemiological settings in sub-Saharan Africa [5], [6], [8], [32], we have conducted a qualitative pilot study to explore context specific determinants of RDT perception among health care workers in Zanzibar in order to improve the understanding of factors influencing adherence to RDTs [33]. However, it should be acknowledged that the health care workers enrolled in our study all received a relatively extensive pre-study training. Moreover, they are all working in an area where other malaria research activities have been conducted in recent years. These factors may have contributed to the high adherence to RDT results, which may not necessarily be representative for the general primary health care worker in Zanzibar.

The incorporation of malaria RDT in IMCI provides an opportunity for improved childhood fever case management in Africa. This study is, to our knowledge, the first scientific evidence from sub-Saharan Africa that RDT can be reliably integrated in IMCI. There was neither any difference in RDT performance nor in adherence to RDT test results in children <5 years of age compared with other age groups. The ability of HRP2 based RDT to reliably rule out malaria infection in a low transmission setting is critical. However, to ensure a sustainable adherence to particularly RDT negative results, health care workers need to be trained and equipped with tools for improved management of non-malarial fevers. A prerequisite for such improved non-malarial fever management is to study the etiology of acute uncomplicated fevers in rural settings of Zanzibar. Such studies, which are ongoing, will in addition provide data on antibiotic requiring bacterial infections among fever patients in Zanzibar. This is critical, since the observed antibiotic prescription rate of 55–65% among RDT negative patients in all age groups as well as RDT positive children <5 years of age (in the form of dual treatment with ACT) probably represents a substantial overuse, which may fuel development and spread of antibiotic resistance.

Our observation of low *P. falciparum* parasite prevalence among fever patients in Zanzibar is coherent with official programmatic data [12]. Interestingly, the highest RDT positivity rate was found in children aged 5–14 years, not in children <5 years. Furthermore, there was an apparent uneven spatial distribution of RDT positivity rate both within and between the study districts. These changing malaria epidemiological patterns, together with the association between the risk of RDT positivity and absence of ITN/LLIN use as well as travel history, need to be considered in future malaria control activities in Zanzibar.

In summary, the sensitivity of HRP-2 based RDT in the hands of primary health care workers compared with both PCR and microscopy for *P. falciparum* case detection was relatively low, whereas adherence to test results with anti-malarial treatment was excellent. Moreover, the results provide evidence that RDT can be reliably integrated in IMCI as a tool for improved childhood fever management. However, the overall relatively low RDT sensitivity highlights the need for improved quality control of RDT use in primary health care facilities, but also for more sensitive point-of-care malaria diagnostic tools in the new epidemiological context of low malaria transmission in Zanzibar.
